# Supplementary material for: MYC regulates ductal-neuroendocrine lineage plasticity in pancreatic ductal adenocarcinoma associated with poor outcome and chemoresistance
Source: Nat Commun. 2017 Nov 23;8:1728. doi: 10.1038/s41467-017-01967-6 (PMC5701042; doi:10.1038/s41467-017-01967-6)
Supplement: Supplementary file 1 — Supplementary Information [file 41467_2017_1967_MOESM1_ESM.pdf]

a

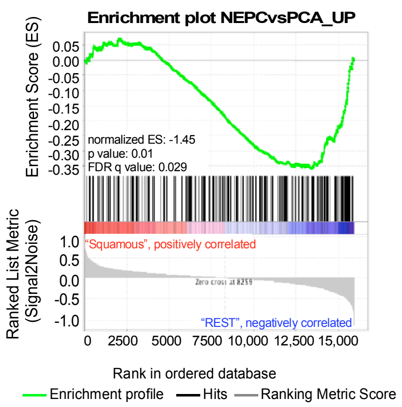

b

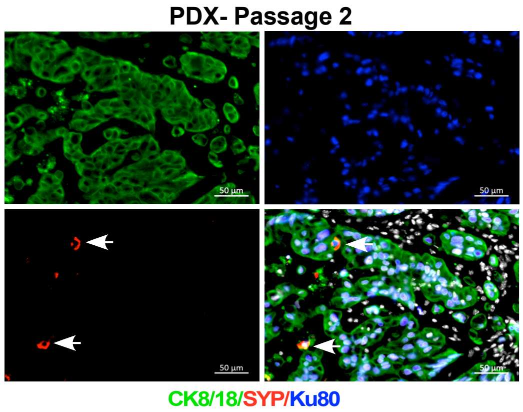

Supplementary Figure 1. NE differentiation in poor outcome PDA and human PDX tissue a. GSEA shows that genes upregulated in PCA vs. NEPC are de-enriched in the squamous subtype **b.** Cells capable of expressing SYP survive passaging in mice in patient-derived xenografts (PDXs). Tissue after two passages in mice was stained with CK8/18, SYP, and the human-specific Ku80 marker. Co-staining cells are marked with white arrows. Scale bars indicate 50 µM.

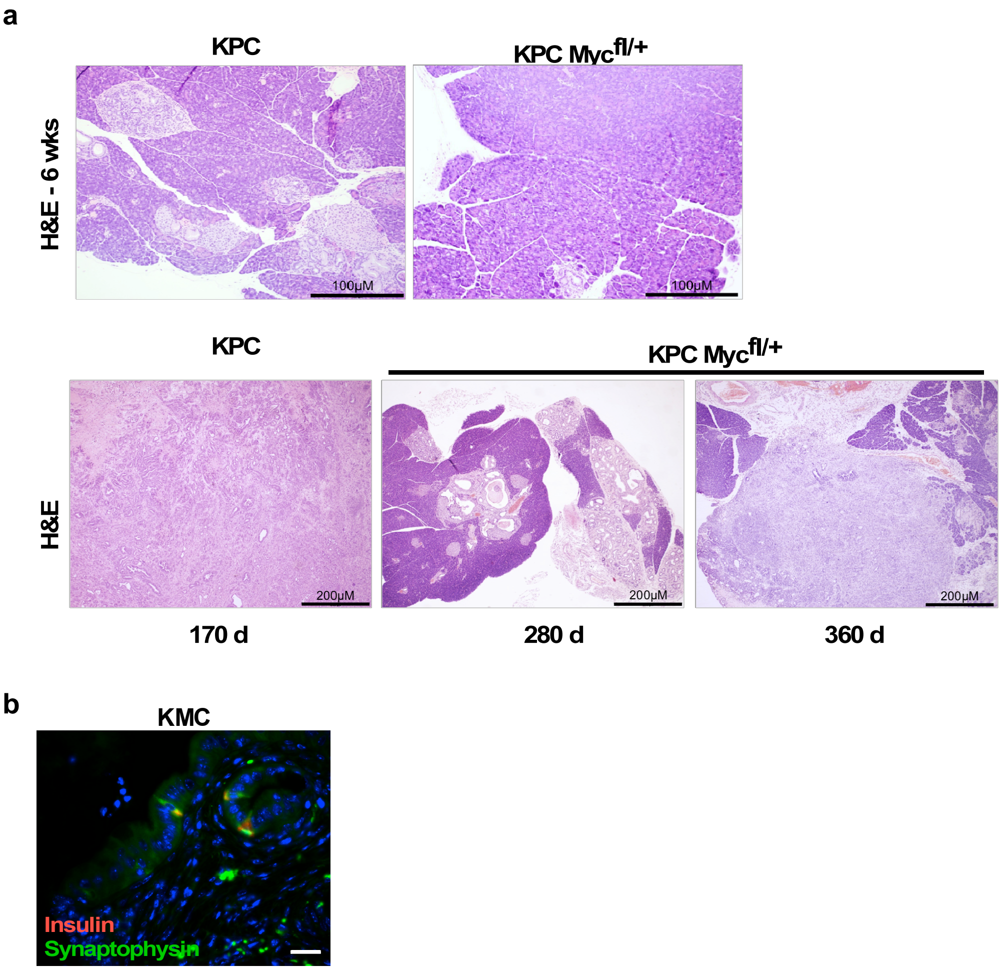

Supplementary Figure 2. MYC regulates ductal-neuroendocrine lineage plasticity
a. Pancreatic tissue from KPC and KPC Myc$^{fl/+}$ mice were harvested at 6 weeks (upper panel) or at end-stage (lower panel) and stained with H&E. Representative images are shown, scale bars indicate either 100 μM or 200 μM. **b.** KMC tumors were stained with Insulin/SYP by IF. A representative image is shown. Scale bars indicate 100 μM.

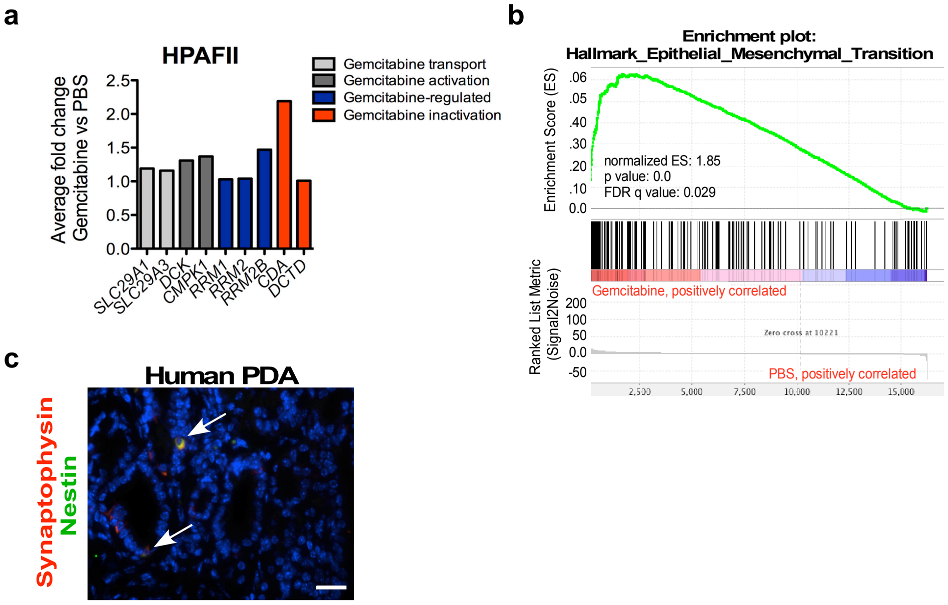

Supplementary Figure 3. Neuroendocrine differentiation and gemcitabine resistance a. HPAFII cells were treated with PBS or gemcitabine and RNA sequencing was performed. Fold change in genes known to be associated with gemcitabine resistance is shown for gemcitabine treated versus PBS-treated HPAFII cells**. b.** GSEA of RNA sequencing data from HPAFII cells treated with gemcitabine versus PBS. **c.** Human PDA samples were stained with Nestin/SYP by IF. A representative image is shown. Co-stained cells are marked with white arrows. Scale bars indicate 100 µM.

Figure 4e - SubQ PDX xenografts, vehicle or Gemcitabine

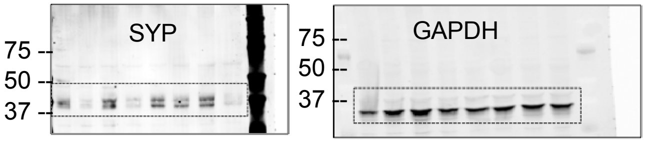

Figure 5a - MiaPaca2 siNT and siMYC triplicates

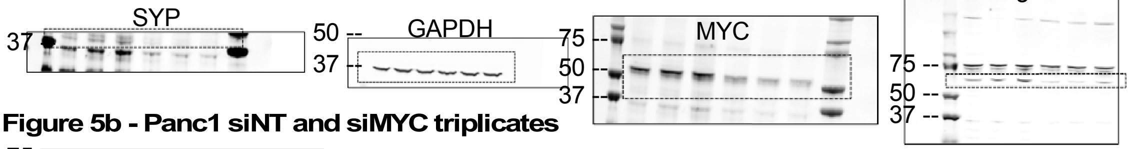

Figure 5b - Panc1 siNT and siMYC triplicates

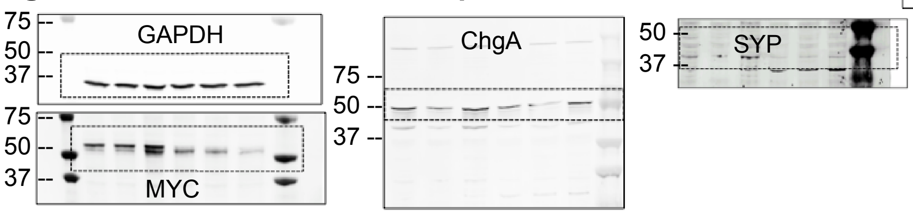

Figure 5c - Capan1 siNT and siMYC triplicates

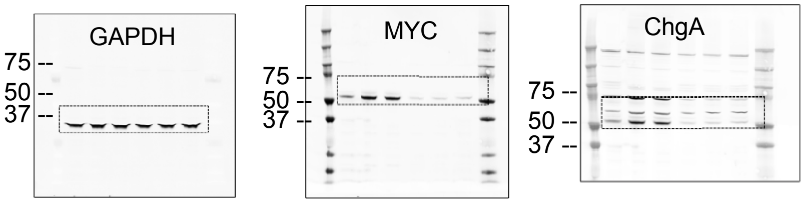

Figure 5d - MiaPaca2, siMYC + Gemcitabine

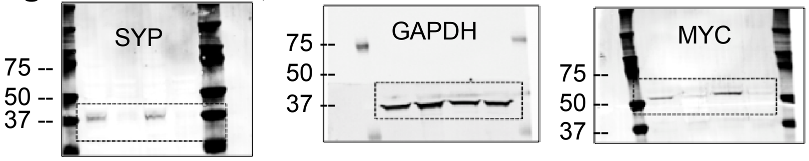

Figure 5d - Capan1, siMYC + Gemcitabine

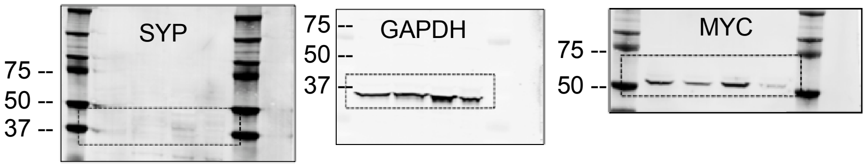

Figure 5d - Panc1, siMYC + Gemcitabine

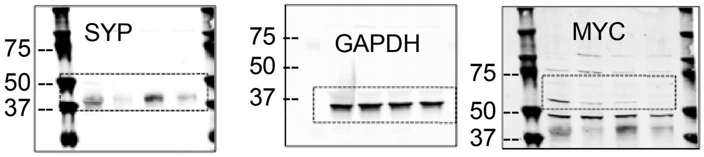

Supplementary Figure 4. Uncropped Western blots

	Low Co-Staining (<5%) n = 10	High Co-Staining (≥5%) n = 6	p-value
Age	67.0 ± 8.8	65.3 ± 7.4	0.704
Grade			0.661
Moderate	50% (5)	66% (4)	
Mod-to-Poor	10% (1)	0.0% (0)	
Poor	40% (4)	33.3% (2)	
Stage			0.221
1A	100% (2)	0% (0)	
1B	75% (3)	25% (1)	
2A	0% (0)	100% (2)	
2B	50% (1)	50% (1)	
3	0% (0)	100% (2)	
LVSI	66.6% (6)	83.3% (5)	0.905
PNI	60% (9)	40% (6)	0.156
R0 Status	100% (10)	100% (6)	1
%PDAc	100% (10)	100% (6)	1
%Gem	90.0% (9)	83.3% (5)	0.319

Supplementary Table 1. Clinical information pertaining to patients from Fig. 1b-c

LVSI = lymphovascular space invasion, PNI = perineural invasion, R0 = absence of residual tumor after surgical resection, PDAc = pancreatic ductal adenocarcinoma, Gem = Gemcitabine

Patient	% CK-SYP Costaining	Tumor Grade	Stage	Documented Recurrence	Days from Diagnosis to Recurrence
1	0.0	Grade III	IIB	regional recurrence	217
2	0.0	Grade III	III	distant recurrence	326
3	0.6	Grade II	IIB		
4	2.0	Grade II	IIB		
5	3.7	Grade III	IIB		
6	4.2	Grade II	IV	distant recurrence	83
7	4.3	Grade III	IIB	distant recurrence	80
8	11.1	Grade II	IIA		
9	11.2	Grade II	IIB	never disease-free	never disease-free
10	19.4	Grade II	IIB	never disease-free	never disease-free

Supplementary Table 2. Clinical information pertaining to patients from second cohort of CK-SYP staining

**Human primers used for qRT-PCR analysis**	**Sequences**
TBP:	Forward 5' – TGCACAGGAGCCAAGAGTGAA – 3'
	Reverse 5' – CACATCACAGCTCCCCACCA – 3'
SNAP25:	Forward 5' – ATGGATGAAAACCTAGAGCAGG – 3'
	Reverse 5' – ACACTTAACCACTTCCCAGC – 3'
SYP:	Forward 5 – AGACCGAGAGTGACCTCAG – 3'
	Reverse 5' – GTCCCCAACTAAGAAGACCT – 3'

Supplementary Table 3. qRT-PCR primer sequences
